# Disruption of GxxxG motifs in pATOM36 impairs biogenesis of the mitochondrial protein translocase of the outer membrane in *Trypanosoma brucei*

**DOI:** 10.1016/j.jbc.2026.113126

**Published:** 2026-05-06

**Authors:** Stephan Berger, Siri Speck, André Schneider, Christoph von Ballmoos

**Affiliations:** 1 Department of Chemistry, Biochemistry and Pharmaceutical Sciences, University of Bern, Bern, Switzerland; 2 Graduate School for Cellular and Biomedical Sciences, University of Bern, Bern, Switzerland

**Keywords:** *Trypanosoma brucei*, mitochondrial protein import, outer mitochondrial membrane protein biogenesis, outer membrane insertase, GxxxG motif, ATOM complex, pATOM36, complementation study

## Abstract

Mitochondrial biogenesis requires efficient import of cytosolically produced proteins and correct segregation of the mitochondrial genome during cytokinesis. In *Trypanosoma brucei*, a parasitic protozoan with a single mitochondrion harboring a single-unit mitochondrial genome, protein import across the outer membrane is mediated by the ATOM complex. An important, yet poorly understood role is played by the integral membrane protein pATOM36 of the outer mitochondrial membrane, which is essential for both ATOM complex assembly and mitochondrial DNA segregation. Here, we combined *in vivo* functional mutational analysis and structural modeling to investigate the function of pATOM36. AlphaFold3-based models predict five highly tilted helices forming a funnel-shaped cavity open toward the cytoplasm, reminiscent of membrane protein insertases. In the model, the protein is sealed towards the mitochondrial intermembrane space by tight helix packing, with conserved GxxxG motifs potentially facilitating these helix-helix interactions. Progressive replacement of these glycines by isoleucines does not affect protein production or correct localization but leads to defective ATOM complex biogenesis and arrest of growth, while mitochondrial DNA segregation is largely unaffected. Based on the predicted structure, these effects can be rationalized by hydrophobic bulking that interferes with associated electrostatic interactions. This hypothesis is supported by experimental mutational analysis of the respective electrostatic interactions in the presence of native GxxxG motifs. Together, our data support the hypothesis that pATOM36 functions as an outer mitochondrial insertase and arose by convergent evolution. The GxxxG motifs, also found in unrelated yeast and human outer membrane insertases, are crucial for protein activity.

Mitochondria are found in virtually all eukaryotes. They originate from a single endosymbiotic event between an archaeal host cell and an α-proteobacterium that subsequently converted into the mitochondrion (1, 2, 3). This process involved extensive loss and transfer of genes from the endosymbiont to the host genome. As a result, more than 95% of all mitochondrial proteins in extant eukaryotes are encoded in the nucleus, synthesized in the cytosol, and finally imported into the organelle. In contrast, only very few but essential genes were retained on the mitochondrial genome. Thus, both protein import and organellar genome inheritance are essential processes for mitochondrial biogenesis (4, 5).

Mitochondrial protein import has been best studied in the yeast *Saccharomyces cerevisiae*. The translocase of the outer mitochondrial membrane (TOM) complex serves as the general entry gate for most mitochondrial proteins. Exceptions are most integral outer mitochondrial membrane (OMM) proteins with a single or with multiple transmembrane helices (TMHs). This group of proteins, which include subunits of the TOM complex, require the mitochondrial import (MIM) complex for their insertion and/or assembly into the OMM. In contrast to the TOM complex, which is highly conserved in nearly all eukaryotes, the MIM complex, composed of single membrane spanning proteins Mim1 and Mim2, has only been found in fungi (Fig. 1*A*) (2, 6, 7). It has recently been shown that the human mitochondrial carrier homolog 2 (MTCH2), an integral OMM protein of the SLC25 family, functions as an insertase for α-helically-anchored OMM proteins, including subunits of the TOM complex (8, 9). Moreover, there is evidence that the MTCH2 paralog MTCH1 also has insertase activity (9). Despite their similar functions, MTCH2 shows no sequence or structural similarity to Mim1 or Mim2, respectively.

**Figure 1:**
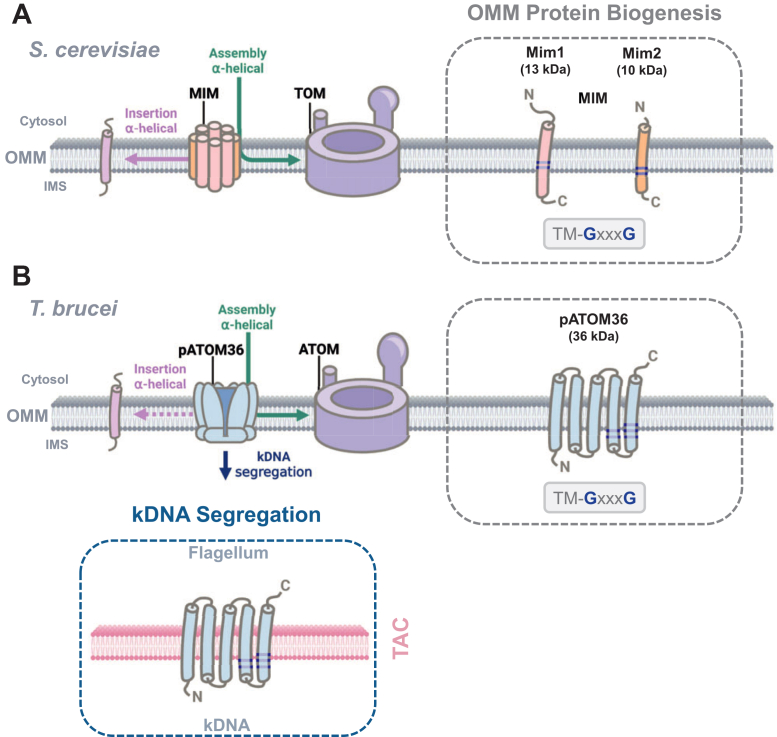
**Outer membrane biogenesis pathways in *S. cerevisiae* and *T. brucei*.***A*, the import machinery of the OMM (*grey*) in *S. cerevisiae* includes the MIM complex, consisting of Mim1 (*pink*) and Mim2 (*orange*) and the TOM-complex (*lavender*). The MIM complex is involved in the assembly of the TOM complex (*turquoise*) as well as in the insertion of a specific subset of α-helical outer mitochondrial membrane proteins (*pink*). Predicted TM-GxxxG helix-helix interaction motifs are depicted in *dark blue*. *B*, the import machinery of the OMM in *T. brucei* includes pATOM36 (*light blue*) and the ATOM complex (*lavender*). pATOM36 is proposed to facilitate the insertion of α-helical membrane proteins (*pink*) and is required to mediate ATOM complex (*turquoise*) assembly. pATOM36 is enriched in TAC region of the OMM (*coral*), where it is required for the correct kDNA segregation (*blue*).

The parasitic protozoan *Trypanosoma brucei* belongs to the eukaryotic supergroup of the Discoba which is only very distantly related to the Opisthokont supergroup that includes *S. cerevisiae* and mammals. This high phylogenetic divergence is reflected by many unique features of *T. brucei*, including the mitochondrial genome inheritance mechanism and the mitochondrial protein import systems (10, 11, 12). In contrast to most eukaryotes, *T. brucei* has a single mitochondrion harboring a single nucleoid genome, termed kinetoplast DNA (kDNA). Segregation of the replicated kDNA into daughter cells depends on the tripartite attachment complex (TAC), which physically links the kDNA across both mitochondrial membranes to the basal body of the flagellum (13, 14, 15). Moreover, *T. brucei* has a diverged TOM complex, termed atypical TOM (ATOM), which, as its yeast counterpart, consists of seven subunits. However, only two of them, ATOM40 and ATOM14, are conserved. They are orthologs of Tom40 and Tom22, respectively (16, 17, 18). The OMM of the *T. brucei* mitochondrion also contains the peripheral ATOM36 (pATOM36), a membrane protein that is required for the biogenesis of a subset of α-helically anchored OMM proteins, including all ATOM subunits except for ATOM40. Intriguingly, pATOM36 is also an essential subunit of the TAC and, therefore, required for kDNA segregation. In line with its dual function, pATOM36 is localized all over the OMM but also enriched in the TAC region (Fig. 1*B*) (5, 19, 20).

Expression of pATOM36 in a yeast ΔMim1/Mim2-deletion strain complements for both the growth and TOM assembly deficiencies observed in these cells. Conversely, the ATOM complex assembly defect observed in pATOM36-depleted trypanosomes is reversed by the simultaneous expression of Mim1 and Mim2. However, the complemented pATOM36 cell line still shows a slow growth phenotype because the MIM complex cannot function as a TAC subunit (21). Furthermore, as in the case of pATOM36, expression of human MTCH1, but not MTCH2, complements the growth defect and TOM assembly deficiencies of the yeast ΔMim1/Mim2-deletion strain (9). These results demonstrate that the essential functions of the yeast MIM complex and the OMM protein biogenesis function of trypanosomal pATOM36 are identical, and the same is likely the case for MTCH1 and MTCH2. Intriguingly, the various proteins in yeast, trypanosomes and humans do not derive from a common ancestor but evolved independently. Thus, the MIM complex, pATOM36, and MTCH1/MTCH2 represent three different solutions to the general problem of how to insert and assemble α-helically anchored mitochondrial OMM proteins. However, it remains unclear which structural elements of pATOM36 are critical for its function. Only C-terminal truncations have been examined so far, with deletion of 75, but not 60, amino acids abolishing function (5). Here, we investigate the functional relevance of this 15-residue stretch by site-directed mutagenesis of pATOM36 and analysis of the resulting effects on its function. Using AlphaFold3-based structural predictions in combination with an *in vivo* complementation system, we performed a mutational analysis of trypanosomal pATOM36. The mutations covered two regions of pATOM36. First, we analyzed the importance of conserved GxxxG motifs in the C-terminal region of pATOM36 that previously have been shown to be essential for ATOM complex biogenesis (5). Interestingly, GxxxG motifs predicted at similar membrane locations are also found in Mim1/Mim2, MTCH1, and MTCH2. Mutations of the GxxxG motifs in Mim1 were shown to impair oligomerization (22), now recognized as assembly of the heteromeric Mim1/Mim2 MIM complex (7). Second, we analyzed charged amino acids predicted in the hydrophilic groove identified in the structural model, which may form a salt bridge network. Transgenic trypanosomes exclusively expressing the different pATOM36 mutants were analyzed for growth, ATOM complex assembly, and defects in kDNA segregation.

## Results

### Structural model of pATOM36

**Figure 2:**
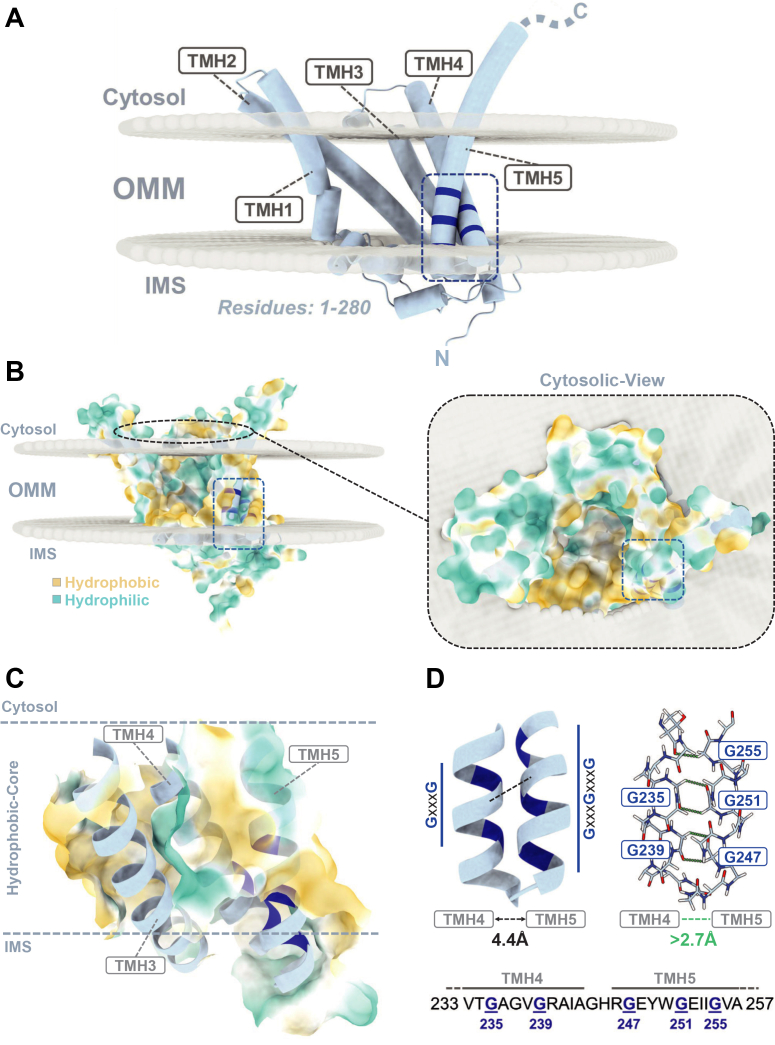
**Structural model of pATOM36 and identification of a transmembrane GxxxG helix–helix interaction motif.***A*, the model of pATOM36 (residues 1–280) embedded in the OMM was generated using AlphaFold3 and oriented with the OPM PPM 3.0 algorithm. The model comprises five highly tilted TMHs (*dark grey*), consistent with a topology in which the C-terminus is exposed to the cytosol and the N-terminus faces the IMS. The position of the single GxxxG motif predicted in TMH4 and the double GxxxG motif in TMH5 are highlighted here and later in *dark blue*. *B*, *Left*: Membrane view of pATOM36 showing hydrophilic (*cyan*) and hydrophobic (*brown*) regions. *Right*: Cytosolic view of the hydrophilic groove within the OMM. *C*, close-up view of the *right* lateral seal formed by the tight packing of TMH3, TMH4, and TMH5, enabled by interaction of the GxxxG motifs. *D*, *Left*: The predicted GxxxG scaffold (*dark blue*) is mediated by opposing residues on TMH4 and TMH5, enabling close interhelical proximity. The distance (*dashed black line*) between the Cα atoms of A236 on TMH4 and A252 on TMH5 is 4.4 Å and is used a proxy for the interhelical distance. *Right*: The scaffold is predicted to be stabilized by five hydrogen bonds (*green*) between the backbone atoms of the glycine residues in TMH4 and TMH5, each with a model-derived interatomic distance of less than 2.7 Å.

A structural model based on the amino acid sequence of pATOM36 was generated using AlphaFold3 (23) and its positioning in the OMM was predicted subsequently using the PPM 3.0 algorithm provided by the OPM database (Fig. 2 *A*) (24). The model predicts pATOM36 to consist of five highly tilted TMHs with a short extramembranous N-terminus and a ∼60 aa stretch at the C-terminus reaching into the IMS and cytoplasm, respectively, in accordance with the experimental localization of the latter (19). Apart from the N- and C-termini, the AlphaFold3 model is predicted with high confidence, reflected by a pTM of 0.83 and a global pLDDT of 83.2 (Fig. S1*A*).

Strikingly, the five predicted TMHs do not form a packed barrel of parallel and vertically oriented α-helices as in many membrane proteins, but show a funnel-like arrangement with a cavity open towards the cytosol and a lateral opening connecting its hydrophilic interior with the surrounding modelled hydrophobic lipid bilayer, as indicated in the hydrophobicity surface representation (Fig. 2*B*). Similar structural features are found in protein insertases of the YidC/Alb3/Oxa1 family (25, 26, 27, 28). This structural architecture is thought to promote membrane protein integration by providing a membrane-embedded hydrophilic groove facilitating the lateral insertion of nascent transmembrane segments into the lipid bilayer (29, 30). The lateral release might be facilitated by amino acids providing a hydrophobic surface that might act as a transition pathway for newly inserted substrate α-helices (Fig. 2*B*, inset, brown colored).

A similar cavity has been identified in the predicted models of human mitochondrial insertases MTCH1 and MTCH2, promoting their α-helical membrane protein insertion activity (8, 9). The structurally analogous groove in the pATOM36 model suggests that a conserved structural framework for substrate engagement and insertion may be maintained across evolutionarily distant species.

In the model, helices three to five are packed together close to the IMS, sealing access to this compartment, in agreement with substrate insertion from the cytosolic side (Fig. 2*C*). Also in this region, along the rim of the two-helix bundle formed by TMH4 and TMH5 in the structural model, a GxxxG-interaction scaffold is observed at the base of the groove (Fig. 2*D*) as detailed in the next paragraph.

### GXXXG motifs on TMH four and five are predicted to enable tight helical packing

The pATOM36 model explains the earlier finding that an ectopically expressed variant with a Δ60 deletion at the C-terminus is viable, while a Δ75 deletion is not (5). The predicted TMH5 is still nearly complete in the Δ60 variant, but is strongly shortened in the Δ75 variant, indicating that the additional 15 amino acids are membrane-embedded in TMH5 in the structural model. Within this 15 aa stretch, the sequence ^247^**G**EYW**G**EII^255^**G** harbors the abovementioned two consecutive GxxxG motifs, with the glycine residues oriented towards TMH4 in the model. Notably, in the model, a third motif on TMH4 with the sequence ^235^**G**AGV^239^**G** is found exactly opposite to the double motif on TMH5, allowing a short distance between TMH4 and TMH5 at this position (Fig. 2*D*, left panel). Indeed, the distances are short enough to allow formation of characteristic weak Cα–H···O hydrogen bonds between glycine residues and backbone carbonyls across the interface, a hallmark of GxxxG-mediated packing (31). As shown in Figure 2*D* (right panel), five interactions with model-derived distances < 2.7 Å form a consecutive ladder along the interface, suggesting that these weak backbone hydrogen bonds may cumulatively contribute to stabilizing the TMH4-TMH5 interaction in pATOM36 within the low-dielectric environment of the membrane.

As a measure of the overall distance between the helices, the Cα–Cα distance between A236 on TMH4 and E252 on TMH5 in the predicted wild-type model was calculated and will be used in the following to assess the impact of the mutations in the respective structural models.

We also predicted structures and membrane localization of Mim1 and Mim2. Both proteins are single-pass membrane proteins whose N-termini are oriented toward the cytosol, while the C-termini reside within the mitochondrial IMS (32, 33). Mim1 and Mim2 are likely to form an oligomeric complex, but the stoichiometry is unknown (33). In Mim1, the present GxxxG motif has been shown to promote oligomerization (22). Notably, our computational analysis indicates that GxxxG motifs in both Mim1 and Mim2 are located within their single TMH towards the IMS (Fig. S2*A*). A similar localization of GxxxG motifs towards the IMS is also found in predicted models of MTCH2 (8) and MTCH1, although not on adjacent helices as in pATOM36 (Fig. S2*B*).

### pATOM36-HA can replace the endogenous pATOM36 in procyclic *T. brucei*

**Figure 3:**
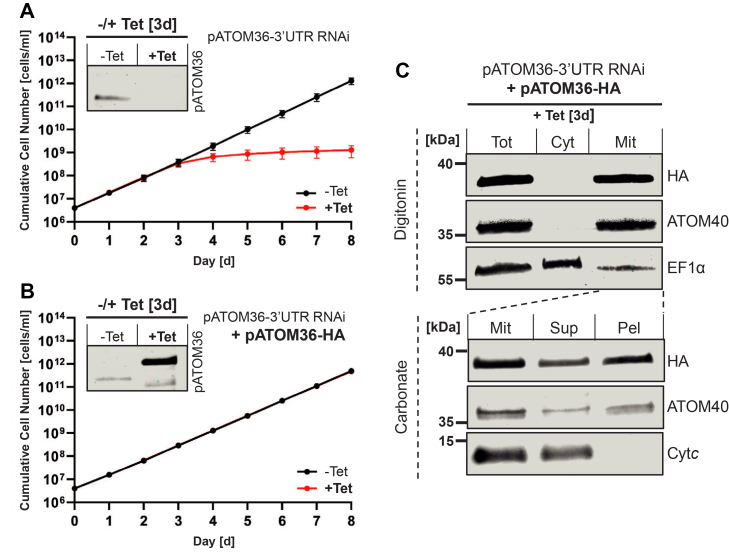
***In vivo* complementation of procyclic *T. brucei* pATOM36 RNAi cells.** Growth analysis of the (*A*) pATOM36–3′UTR RNAi cell line and (*B*) the supplemental C-terminal 3xHA-tagged wild-type pATOM36 (pATOM36-HA) transfection into the 3′UTR pATOM36 RNAi (pATOM36–3′UTR RNAi + pATOM36-HA) cell line in the absence (*black*) and in the presence (*red*) of tetracycline (Tet). (Inset) Knockdown efficiency and simultaneous expression of pATOM36-HA were assessed in crude mitochondria using an pATOM36-specific antibody. *C*, fractionation analysis of pATOM36-HA after 3 days of Tet induction. *Top*: Western blots of total (Tot), digitonin-extracted cytosolic (Cyt), and crude mitochondrial (Mit) fractions. ATOM40 and EF1α serve as mitochondrial and cytosolic markers, respectively. *Bottom*: Western blots of alkaline carbonate extractions from the same mitochondria-enriched fraction (Mit). The resulting pellet (Pel), containing integral membrane proteins, and the supernatant (Sup), containing soluble and peripheral membrane proteins. ATOM40 and cytochrome *c* (Cyt*c*) serve as markers for integral and peripheral membrane proteins, respectively.

pATOM36 is essential for the normal growth of both procyclic and bloodstream form of *T. brucei*. Using a previously established RNAi cell line targeting the 3′UTR of the pATOM36 mRNA (pATOM36–3′UTR RNAi), we showed that depletion of pATOM36 caused a growth arrest 3 days after tetracycline induction (Fig. 3*A*). Ectopic overexpression of a C-terminally triple HA-tagged pATOM36 variant (pATOM36-HA) from an mRNA with an alternative 3′UTR in the same cell line completely restored growth. This demonstrates that pATOM36-HA can fully complement all essential functions of pATOM36 (Fig. 3*B*). In line with these results, digitonin-based cell fractionation shows that the pATOM36-HA co-fractionates with the crude mitochondrial fraction, while alkaline carbonate extraction at pH 11.5 confirms its integration into the mitochondrial membrane (Fig. 3*C*) (34).

**Figure 4:**
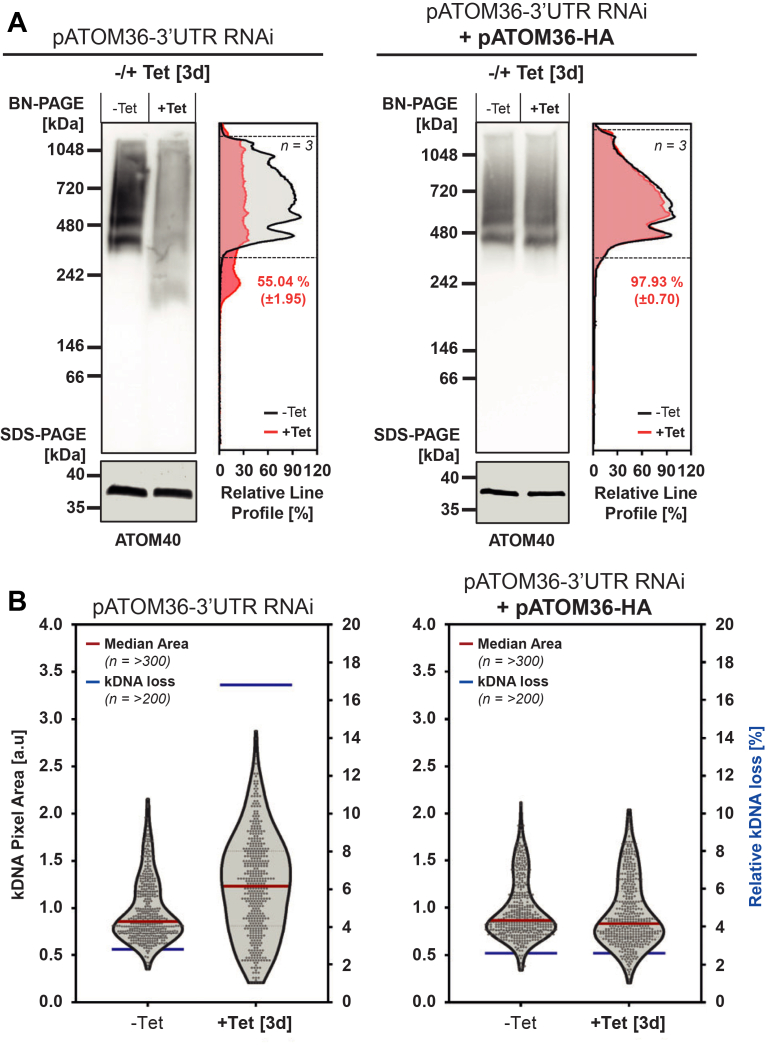
**Analysis of ATOM complex assembly and in kDNA segregation.***A*, ATOM complex assembly in pATOM36-3′UTR RNAi and pATOM36-3′UTR RNAi + pATOM36-HA cell lines was assessed in mitochondrial membrane extracts by BN-PAGE, followed by immunoblotting with an ATOM40-specific antibody in tetracycline-induced (+Tet) and non-induced (−Tet) cells after 3 days. Detection of ATOM40 served as loading control. Molecular weight markers (kDa) are shown as *dashes*. Pixel line profiles were generated in ImageJ, and mean pixel intensity within the region containing the major ATOM40-containing complexes (*dashed box*) was quantified. Relative signal changes in the +Tet condition (*red*) were calculated from biological triplicates. *B*, mitochondrial kDNA segregation was assessed by measuring kDNA area in −Tet cells and +Tet cells after 3 days of induction. Pixel areas of individual DAPI-stained kDNA networks were quantified using a semi-automated macro in ImageJ. The *red line* marks the median area (n = 300). kDNA-loss phenotypes, were evaluated manually by overlaying DAPI and brightfield images, and the *blue line* indicates the relative extent of kDNA loss (n = 200).

On a mechanistic level, pATOM36 is required for ATOM complex assembly, as in its absence, except for ATOM40, many of its subunits get depleted (16). In uninduced cells, BN-PAGE resolves up to four distinct ATOM40-containing complexes with molecular weights ranging from approximately 400 to 700 kDa. After induction of the pATOM36-3′UTR RNAi cell line, these complexes are reduced in size, and their amount is depleted to approx. 55% of that in non-induced cells (Fig. 4*A*, left panel). In line with the restoration of growth after expression of pATOM36-HA in the same cell line (Fig. 3*A*), assembly of the ATOM complex is also complemented to wild-type levels (Fig. 4*A*, right panels).

pATOM36 is not only essential for OMM protein biogenesis but is also a subunit of the TAC complex (5, 35, 36). Lack of an intact TAC complex is monitored by an abnormal kDNA segregation phenotype. In this phenotype, failure of kDNA transfer into daughter cells leads to over-replication of kDNAs in some cells and kDNA loss in other cells. In addition, very small kDNAs are also observed (5). In summary, this yields a cell culture with heterogeneous kDNA size distribution, in which the fraction of kDNA-lacking cells will continually increase during RNAi-mediated depletion of a given TAC subunit. This phenotype is similar in cells lacking pATOM36 as well as other TAC complex proteins such as TAC40, TAC42, TAC60, TAC65, p197 or p166 (5, 15, 37, 38, 39, 40). The kDNA segregation phenotype is monitored by fluorescence microscopy of DAPI-stained cells and digital image analysis, which allows for scoring the size distribution of the kDNAs. The left panel of Figure 4*B* shows that segregation defects of the kDNAs are evident in the induced pATOM36-3′UTR RNAi cell line. Comparison of non-induced and three-day-induced pATOM36-3′UTR RNAi cells reveals an increase in median kDNA size in cells containing kDNA, accompanied by a higher frequency of kDNA loss (Fig. 4*B*, left). After add-back of pATOM36-HA, however, no significant differences in kDNA size and distribution are observed (Fig. 4*B*, right).

In summary, the results show that pATOM36-HA localizes correctly and is fully functional in our *in vivo* system.

### Functional consequences of GxxxG motif mutations in pATOM36

**Figure 5:**
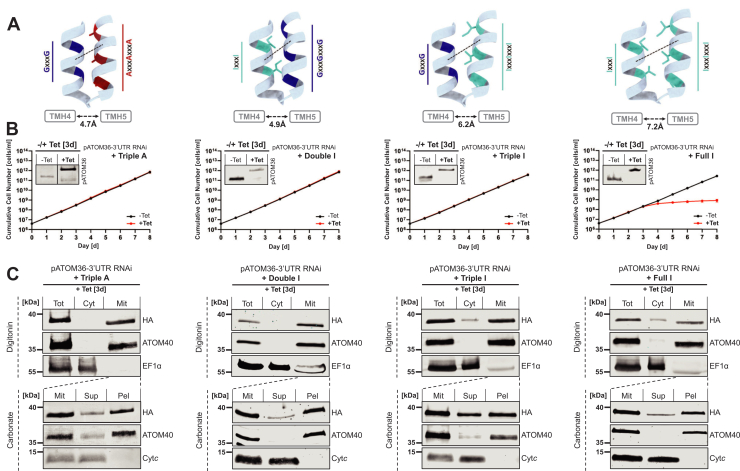
**Growth and protein localization analysis of pATOM36 variants with modifications in the GxxxG scaffold.***A*, AlphaFold3 models of pATOM36 variants; Triple A (G247A, G251A, G255A), Double I (G235I, G239I), Triple I (G247I, G251I, G255I), and Full I (G235I, G239I, G247I, G251I, G255I). Glycines are shown in *dark blue*, alanines in *dark red*, and isoleucines in *cyan*. Also shown is the model-derived distance between the Cα atoms of A236 (TMH4) and E252 (TMH5) (*dashed black lines*). *B*, growth analysis of -Tet non-induced and +Tet induced cell lines expressing C-terminal 3×HA-tagged pATOM36 variants, with corresponding western blots of crude mitochondria confirming Tet-inducible pATOM36–3′UTR RNAi and simultaneous overexpression of the respective variant. *C*, fractionation analysis of pATOM36-HA variants after 3 days of Tet induction. *Top*: Digitonin fractionation western blots of total (Tot), cytosolic (Cyt), and crude mitochondrial (Mit) fractions. ATOM40 and EF-1α serve as mitochondrial and cytosolic markers. *Bottom*: Western blot analysis of alkaline carbonate extractions from the same crude mitochondrial fraction (Mit). Integral membrane proteins remain in the pellet (Pel) and soluble/peripheral proteins in the supernatant (Sup). ATOM40 and Cyt*c* mark integral and peripheral membrane proteins, respectively.

To investigate the functional importance of the GxxxG motifs predicted in TMH4 and TMH5 of pATOM36, we created a series of cell lines expressing pATOM36 variants, in which the conserved glycines in the GxxxG motifs were substituted with either alanines or isoleucines. In parallel, AlphaFold3 models of the respective mutants (Fig. S3) were used to calculate the interhelical Cα-Cα distance between A236 on TMH4 and E252 on TMH5 in the predicted structures to evaluate the impact of the mutations on helical packing. These model-derived calculations suggest that the incorporation of amino acids with larger side chains increases the spatial separation between the two evaluated residues. Being 4.4 Å in the wild-type structural model, introduction of three alanines in the Triple A model (G247A, G251A, G255A) increased the distance to 4.7 Å. The distance further increased to 4.9 Å and 6.2 Å in the Double I (G235I, G239I) and Triple I (G247I, G251I, G255I) model, respectively, and was maximal at 7.2 Å in the Full I (G235I, G239I, G247I, G251I, G255I) model, where all glycines are replaced by isoleucine (Fig. 5*A*).

To test how the mutant pATOM36 variants, all of which were C-terminally tagged with triple HA, affect the function of pATOM36 *in vivo*, we expressed them in the background of the pATOM36-3′UTR cell line. Neither the exclusive expression of the Triple A, the Double I, nor the Triple I mutants affected growth. The Full I variant, however, caused a growth arrest starting 3 days following tetracycline addition (Fig. 5*B*), indicating that the protein is not functional. Depletion of the endogenous pATOM36 as well as expression and mitochondrial localization of the corresponding pATOM36 mutants was confirmed by digitonin-based cell fractionations (Fig. 5*B*). Correct localization of all variants into the OMM was confirmed using alkaline carbonate extraction of the mitochondrial fraction (Fig. 5*C*). Finally, immunofluorescence microscopy shows that all variants co-localize with ATOM40, the central beta barrel subunit of the ATOM complex that is not dependent on pATOM36 for insertion into the OMM (Fig. S4) (5). Strikingly, ablation of pATOM36 leads to a more condensed mitochondrial morphology, similar to previously observed ATOM19-depleted cells, which display morphological changes along with distinct defects in ATOM complex stability and assembly (41, 42, 43) (Fig. S4, top panel). This phenotype is restored in all viable variants, but persists in the Full I variant. Importantly, however, the microscopy data confirm co-localization of the Full I mutant with ATOM40 in the OMM.

Together, these data strongly indicate that all pATOM36 variants are properly folded and localized, as orphaned OMM proteins are targeted for extraction and degradation by the Msp1 quality control pathway (44).

**Figure 6:**
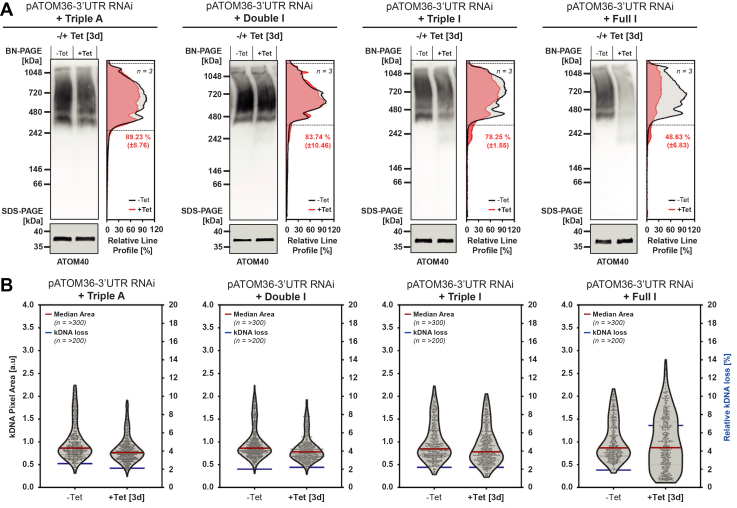
**Impact of pATOM36 GxxxG scaffold modifications on ATOM complex assembly and kDNA segregation.***A*, ATOM complex assembly in variant cell lines was analyzed by BN-PAGE of mitochondrial membrane extracts, followed by immunoblotting with an ATOM40-specific antibody in non-induced (−Tet) and tetracycline-induced (+Tet) cells after 3 days. Detection of ATOM40 served as loading control. Molecular weight markers (kDa) are indicated by dashes. Pixel line profiles are generated with ImageJ, and mean intensity within the major ATOM40-containing complexes region (*dashed black box*) is quantified. Relative intensity changes in the +Tet condition (*red*) are calculated from biological triplicates. For each variant, n = 3, and the mean intensity of the major ATOM40-containing complex region is expressed as a percentage ± SD. *B*, kDNA segregation was evaluated by measuring kDNA area in −Tet cells and + Tet cells after 3 days of induction. Pixel areas of individual DAPI-stained kDNA networks were quantified using ImageJ. The *red line* indicates the median kDNA area (n = 300). kDNA-loss phenotypes were assessed manually from microscopic images, with the *blue line* showing the relative extent of kDNA loss (n = 200).

Next, we analyzed ATOM complex assembly in the mutant cell lines. BN-PAGE followed by Western blotting using an α-ATOM40 antibody of cell extracts from the Triple A and Double I variants revealed no impairment of ATOM complex assembly (Fig. 6*A*), similar to unmutated pATOM36-HA (Fig. 4*A*, right). In the Triple I variant, ATOM assembly is not significantly impaired; however, a weak signal is detected for ATOM40-containing complexes of ∼200 kDa, which is also observed in the induced pATOM36-3′UTR RNAi cell line (Fig. 4*A*, left). In contrast, the Full I variant completely fails to assemble the ATOM complex, reflected by an approx. 50% intensity reduction similar to what is observed in pATOM36-depleted cells (Fig. 4*A*, left).

The same mitochondrial extracts were also analyzed for the putative pATOM36 substrates ATOM19 and ATOM46 (5). As depicted in Fig. S5, only the Full I variant shows complete downregulation of ATOM19 and reduced levels of ATOM46 as observed in the RNAi cell line, while all other variants complemented this effect similar to the pATOM36-HA variant. This is consistent with depletion studies of core ATOM components, where loss of α-helical subunits such as ATOM14 leads to reduced ATOM19 levels, reflecting their interdependence (16, 18). In contrast, the receptor subunit ATOM46 has been shown to be less dependent on the α-helical core components (16), which may explain its comparatively moderate reduction. The strong down regulation of ATOM19 complex found in the Full I variant offers an explanation for the altered mitochondrial morphology described above.

Finally, we also analyzed how the mutant pATOM36 variants affect kDNA segregation. As depicted in Figure 6*B*, Triple A, Double I, and Triple I variants do not exhibit kDNA loss or missegregation, and kDNA size distributions remain comparable to those observed under non-induced conditions (Fig. 6*B*, left). The results for the Full I variant were similar, although its kDNA loss (∼7%) was elevated from the background levels (∼2–3%) observed in uninduced and induced RNAi cell lines of the other variants, but much less than in the induced parental pATOM36-3′UTR RNAi cell line (∼17%) (Figs. 4*B*, left, S6). The kDNA size distribution of the Full I variant was broader than for the other variants, but the median of kDNA size was, unlike in the pATOM36-3′UTR RNAi cell line, not affected. Such a phenotype (mild kDNA loss, broad distribution, but no overreplication) has also been found when ATOM40 was depleted, where formation of an ATOM complex is not possible, but all components of the TAC complex are present (5). Thus, while we cannot exclude that the Full I variant is to some extent deficient for kDNA segregation, the observed phenotype can be explained as a consequence of an indirect effect of the ATOM complex assembly deficiency of the Full I variant. Analogously, ATOM19 depletion, which is downregulated in the Full I variant, has been shown to induce mild kDNA loss (43).

Taken together, the results indicate that bulkier amino acid side chains, such as methyl or sec-butyl groups, which increase steric hindrance within the GxxxG scaffold, do not affect pATOM36 function. Severe impairment of ATOM assembly was observed only in the Full I variant, where the computationally predicted spatial distance between TMH4 and TMH5 was increased to 7.2 Å.

### Identification of a putative salt bridge network enabled by the GxxxG scaffold

**Figure 7:**
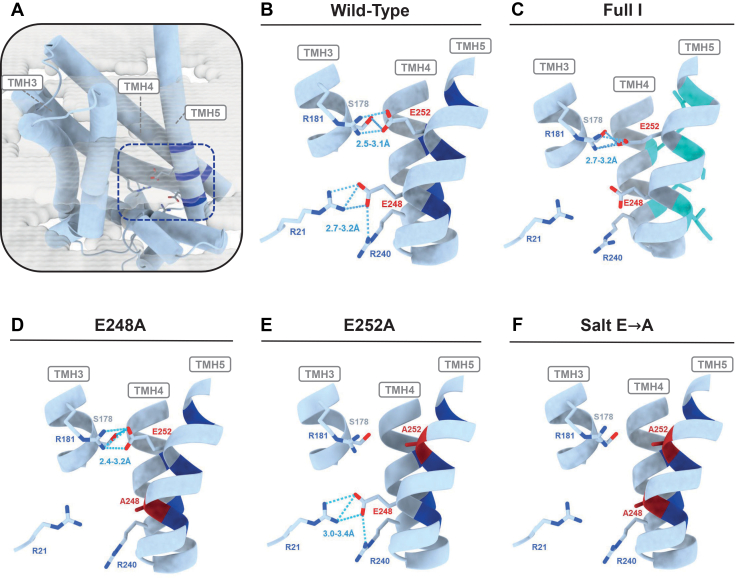
**Identification and organization of a predicted electrostatic interaction network within the GxxxG scaffold.***A*, AlphaFold3 model of wild-type pATOM36 (*light blue*) embedded in the OMM (*gray*), with the position of the salt-bridge network indicated (*dashed box*). *B*, organization of the predicted electrostatic interaction network formed by E248 and E252 (*red*) on TMH5 reveals two distinct clusters: Cluster one includes putative electrostatic interactions (*dashed blue lines*) between E248 and R240 (*blue*) on TMH4, as well as R21 (*blue*). Cluster two comprises potential electrostatic interactions between E252 and R181 (*blue*) and S178 (*light blue*) both on TMH3. Blue distance labels denote the model-derived, computationally determined minimum and maximum distances among all possible interactions. Distance analysis is based on structural models of pATOM36 variants. *C–F*, distance analysis like 7B, from structural models of the indicated variants were used. Introduced isoleucine and alanine residues are shown in *cyan* and *red*, respectively.

Based on the experimental results obtained above, we compared the predicted structures of wild-type pATOM36 and the Full I variant in more detail. We considered whether the isoleucine-induced structural perturbations could have secondary consequences, including the disruption of other stabilizing interactions such as van der Waals forces, electrostatic interactions, and hydrogen bonding (45). Prominently, the computational analysis suggests the presence of a putative salt bridge network within the GxxxG double motif predicted on TMH5, organized into two distinct clusters (Fig. 7*A*). Salt bridges in proteins typically show heavy-atom (*e.g.* N or O) distances of 2.7 to 3.2 Å. (46, 47). The first predicted cluster consists of interactions between E248 on TMH5 and R240 on TMH4, as well as R21, which is part of a short horizontal helix located at the interface between the membrane and the IMS. The second predicted cluster involves the fully membrane-embedded E252 on TMH5 forming interactions with R181 and S178 on TMH3. The putative interaction between E252 and S178 would be a hydrogen bond, with S178 acting as H-donor. The predicted interactions are shown in Figure 7*B*, with distances extracted from the computational model.

Analysis of the putative salt bridge network in the Full I pATOM36 structural model indicates that the sterically bulkier sec-butyl side chains of isoleucine do not only increase spatial separation between TMH4 and TMH5, but also alter the predicted interaction pattern within the salt bridge network. The model predicts loss of cluster I involving E248, increasing the distances to R21 and R240. The second cluster is also predicted to undergo conformational changes, with the orientation of the E252 side chain rotated by ∼90°, albeit without significantly increasing its distances to R181 and S178 (Fig. 7*C*).

Noteworthy, the involved residues E248, R21, R181, and R240 are conserved across the Trypanosomatidae. Residue E252, while conserved in the genus *Trypanosoma*, is not fully conserved in all trypanosomatids, but, is, for example, replaced by asparagine, which might also participate in hydrogen bonding (Fig. S7)

Taken together, these considerations based on the computational model of pATOM36 support the hypothesis that the close spatial arrangement of the predicted three-helix bundle formed by TMH3, 4, and five might be relevant for secondary stabilizing interactions such as the network described.

### Functional consequences of mutations within the putative salt bridge network

**Figure 8:**
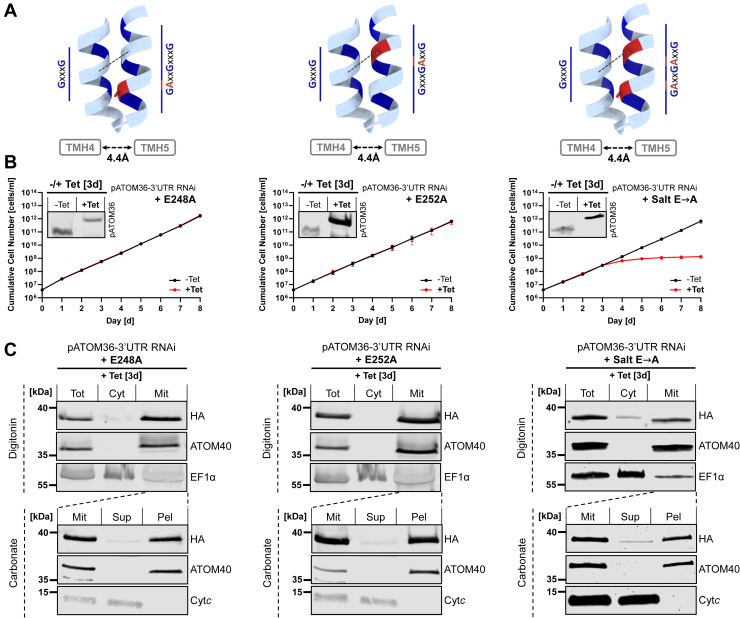
**Growth and protein localization analysis of salt-bridge network variants.***A*, AlphaFold3 models of pATOM36 salt-bridge variants; E248A, E252A, and the double mutant Salt E→A (E248A, E252A). Glycine residues are highlighted in *dark blue*, and alanines in *dark red*. Also shown is the model-derived distance between the Cα atoms of A236 (TMH4) and E252 (TMH5) (*dashed black lines*). *B*, growth analysis of -Tet and +Tet cell lines expressing C-terminal 3×HA-tagged pATOM36 variants, with corresponding western blots of crude mitochondria confirming Tet-inducible pATOM36–3′UTR RNAi and simultaneous overexpression of the respective variant. *C*, fractionation analysis of pATOM36-HA variants after 3 days of Tet induction. *Top*: Digitonin fractionation western blots of total (Tot), cytosolic (Cyt), and crude mitochondrial (Mit) fractions. ATOM40 and EF-1α serve as mitochondrial and cytosolic markers. *Bottom*: Western blot analysis of alkaline carbonate extractions from the same crude mitochondrial fraction (Mit). Integral membrane proteins remain in the pellet (Pel) and soluble/peripheral proteins in the supernatant (Sup). ATOM40 and Cyt*c* mark integral and peripheral membrane proteins, respectively.

To test the *in vivo* importance of the putative salt bridge network on pATOM36 activity, strains exclusively expressing pATOM36 variants either containing the E248A, the E252A, or both substitutions (Salt E→A variant) were created. The mutants were designed to prevent the formation of each cluster of the predicted salt bridge network separately or of both together, respectively (Fig. 7, *D*–*F*). These mutations are not predicted to change the model-derived Cα–Cα distance between A236 on TMH4 and E/A252 on TMH5, as observed in the Full I structural model (Fig. 8*A*).

All three variants were analyzed in the procyclic *T. brucei in vivo* system as described above. After tetracycline addition, endogenous pATOM36 was suppressed, and all tagged variants were expressed. While both single variants grew like wild-type, the Salt E→A variant showed a growth arrest starting 3 days after tetracycline induction (Fig. 8*B*), indicating that the expressed protein is not functional. Digitonin-based cell fractionation reveals that all pATOM36 co-fractionate with the crude mitochondrial fraction. Subsequent sodium carbonate extraction confirms their integration into the mitochondrial membrane (Fig. 8*C*). Immunofluorescence microscopy shows that all variants co-localize with the OMM marker ATOM40, but only the two single mutants were able to restore the condensed mitochondrial network phenotype observed in RNAi cells, the Full I and the Salt E→A variant. (Fig. S4).

**Figure 9:**
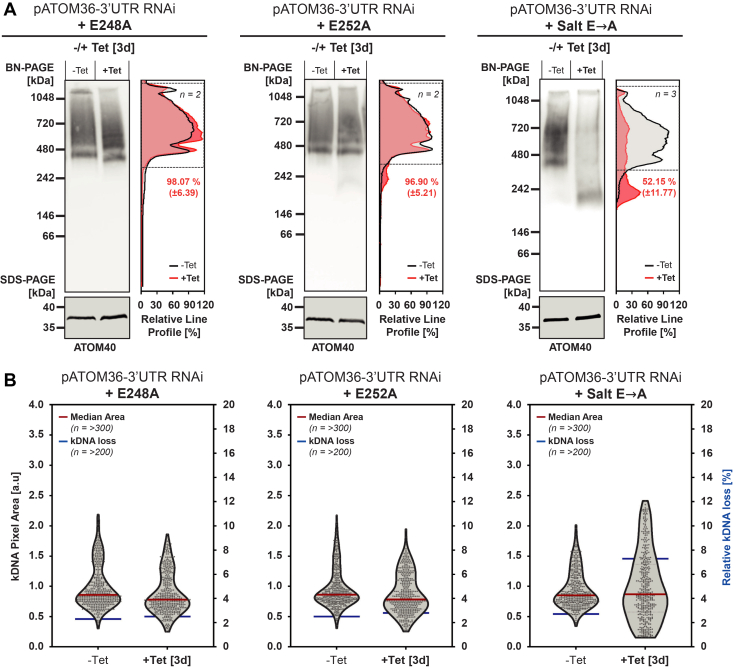
**Impact of pATOM36 salt-bridge network variants on ATOM complex assembly and kDNA segregation.***A*, ATOM complex assembly in variant cell lines was assessed by BN-PAGE of mitochondrial membrane extracts and immunoblotting with an ATOM40-specific antibody in −Tet and +Tet cells after 3 days. ATOM40 blots verified equal loading. Pixel line profiles were generated in ImageJ, and mean intensity within the major ATOM40-containing complexes (*dashed box*) was quantified. Relative intensity changes in +Tet cells (*red*) were calculated from biological triplicates/duplicates. For each variant, the mean intensity of the major region is shown as % ± SD (n = 2 for E248 and E252 variants, n = 3 for Salt E→A). *B*, kDNA segregation was analyzed by measuring kDNA area in −Tet and +Tet cells after 3 days. Pixel areas of individual DAPI-stained kDNA networks were quantified in ImageJ, with the *red line* indicating the median area (n = 300). kDNA-loss phenotypes were evaluated manually from microscopy images, and the *blue line* denotes the relative extent of kDNA loss (n = 200).

Figure 9 shows that ATOM complex assembly was not affected in the single point mutations, although trace amounts of the ATOM40-containing complexes at ∼200 kDa are detected in the E252A variant (Fig. 9*A*, left). In contrast, the Salt E→A double variant fails to assemble the ATOM complex, as reflected by a marked reduction of ATOM40-containing complex intensities to approx. 50% and a pronounced accumulation of the ∼200 kDa ATOM40-containing complex (Fig. 9*A*, right). This corroborates with the failure of this variant to restore the condensed mitochondrial network observed in immunofluorescence. This defect is accompanied by complete downregulation of ATOM19 and reduced levels of ATOM46, like it is observed in the Full I variant (Fig. S5). Analysis of TAC function of pATOM36 reveals that the single-point mutations E248A and E252A do not affect kDNA segregation (Fig. 9*B*, left). In contrast, the Salt E→A variant exhibits a phenotype closely resembling that of the Full I variant, where kDNA loss (∼7%) was above background levels, and the kDNA size distribution was broader. However, the median of the kDNA sizes did not change. As for the Full I variant, these phenotypes could be an indirect effect of the ATOM complex assembly defect (Fig. 9*B*, right).

Taken together, these results suggest that the predicted salt bridge clusters enabled by the GxxxG double motif play an important role in pATOM36 activity. Removal of individual salt bridges does neither affect ATOM complex assembly nor kDNA segregation. In contrast, disruption of the entire predicted salt bridge cluster in the double mutant strongly affects ATOM complex assembly. The same is observed in the Full I model, in which formation of the salt bridge network is prevented by an increased interhelical distance between TMH4 and TMH5.

## Discussion

pATOM36 is an essential, trypanosomatid-specific OMM protein with a dual role. It is required for assembly of the ATOM complex and it ensures kDNA segregation as a TAC subunit (14). pATOM36 is a functional analog of yeast Mim1/Mim2, and *vice versa*, representing an example for convergent evolution of an OMM protein biogenesis pathway (5, 21). While pATOM36 mediates ATOM complex assembly, it has not been directly shown that it mediates OMM insertion of α-helically anchored ATOM subunits. However, given its ability to complement a yeast Mim1/Mim2 deletion strain (21) and because it mediates insertion of the integral OMM protein POMP10, this is very likely (20).

Here, we have used AlphaFold3 and OPM PPM 3.0 to build a structural model of pATOM36. The prediction is of high quality, with particularly high confidence scores within the transmembrane region. The highly tilted arrangement of the five predicted TMHs forms a hydrophilic cavity accessible from the cytosol that is laterally open to the hydrophobic membrane core and, reminiscent of protein insertases of the YidC family (28). Within the predicted structure, three conserved GxxxG motifs on TMH4 and TMH5 are identified that show a tight interhelical packing towards the IMS.

The model predicts that the double GxxxG motif on TMH5 and the corresponding motif on TMH4 lie directly opposite each other, positioning the two helices in close apposition. GxxxG motifs have been shown to mediate intermolecular interactions of neighboring subunits of membrane protein complexes (48, 49). While pATOM36 forms a homo-oligomer of ∼140 kDa (21), the positions of the GxxxG motifs in the predicted structure suggest that they mediate intramolecular interactions and thus are unlikely to contribute to pATOM36 oligomerization.

Interestingly, predicted transmembrane GxxxG motifs situated close to the IMS are also found in Mim1 and Mim2 from yeast, as well as in the predicted structures of MTCH1 and MTCH2. For Mim1 and Mim2, which have a single TMH each and form the hetero-oligomeric MIM complex of unknown stoichiometry (7), the GxxxG motif in Mim1 has been proposed to be implicated in oligomerization (22), suggesting that these motifs may be involved in interhelical interactions.

**Figure 10:**
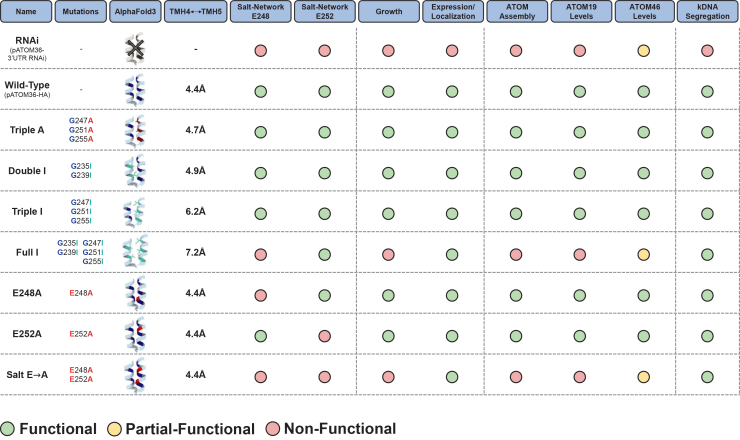
**Overview of the pATOM36 variant cell lines investigated in this work.** Schematic summary of results obtained from individual pATOM36 variant cell lines and the corresponding computational models. Variants exhibiting functional behaviour under the tested conditions are indicated in *green*, whereas variants that are non-functional under the same conditions are shown in *red*.

Our mutational analysis, summarized in Figure 10, suggests that, despite their conservation in Trypanosomatidae, the GxxxG motifs are relatively robust towards amino acid variations. Neither replacement of the three glycines of the double motif on TMH5 with either three alanines or isoleucines, nor the replacement of the two glycines with isoleucines in the motif on TMH4 had an impact on pATOM36 function. In hindsight, this is not too surprising, as the replaced amino acids are apolar and still allow for a tight packing. Strikingly, when all five glycines of the motifs were replaced by isoleucines, cells were unable to grow and ATOM complex assembly was abolished. Such cumulative substitution from the smallest to a bulky hydrophobic residue is likely to affect helix packing, either through hydrophobic bulking or local structural perturbation. While we consider hydrophobic bulking leading to disruption of a putative salt network as a potential mode of action here, the preserved expression levels, correct OMM localization and immunofluorescence data indicate that all variants are stably expressed and do not exhibit gross defects in topology.

A subsequent analysis of the AlphaFold3-based models prompted us to experimentally investigate the importance of two predicted salt bridges that are affected in the predicted Full I structure, where hydrophobic bulking increased the distance of interacting charged residues. Consistent with our hypothesis, we found that removal of the two central acidic residues caused the same phenotype as is seen in the Full I variant, while single mutations behaved normally. In line with these results, it was previously shown that deletion of the 75 C-terminal amino acids of pATOM36 (ΔC75), which removes the GxxxG motifs in TMH5 as well as E248 and E252, shows the same phenotype as the Full I and the Salt E→A variants. However, deletion of the 60 C-terminal amino acids, corresponding to the cytosolically exposed C-terminus, where the GxxxG motifs and E248 and E252 are retained, did not affect either of the two pATOM36 functions (5).

We attribute the lack of growth of the Full I and Salt E→A variants (and of ΔC75) predominantly to the ATOM complex assembly defect, which is sufficient to induce growth arrest as shown earlier (5, 16, 18). While not being a stoichiometric subunit of the complex itself, pATOM36 is required for successful OMM insertion of α-helical ATOM subunits, including the here tested ATOM19 and ATOM46. In the absence of proper insertion and assembly, these subunits were shown to be degraded by the cytosolic proteasome in a pathway linked to Msp1 (44). The detected absence of ATOM19 in the two nonviable variants has twofold consequences. First, the observed growth defect could be a direct consequence of the failed insertion of ATOM19, which is essential for growth (18, 43). Second, direct ablation of ATOM19 also led to kDNA loss as observed for the two non-viable mutants, despite an intact TAC complex (43). Taken together, the impact of the Full I and Salt E→A variants on ATOM complex assembly and pATOM19 is sufficient to explain the growth phenotype, but the additional contribution of the observed kDNA loss phenotype cannot be fully excluded.

The presented structural model, with its distinct arrangement of helices with a laterally open cavity that is reminiscent of the YidC family of insertases, supports this hypothesis (28). The convergently evolved, broadly similar architecture of pATOM36 and YidC suggests a conceptually similar way of membrane protein insertion. Thus, we can conclude from our experiments that the predicted spatial proximity of TMHs 3, four and five close to the IMS is crucial for the insertase function of pATOM36. Moreover, both the GxxxG motifs as well as the salt bridge network are required for this tight packing, as removing either of the two abolishes the function of pATOM36 in ATOM complex assembly. It is noteworthy that, according to the model, only one of the predicted salt bridges (E248 to R21 and R240) lies within the hydrophobic core of the membrane, whereas the other (E252 to R181) is positioned outside, based on membrane insertion predictions using the OPM PPM 3.0 algorithm (24). Since the single mutant (E248A) is unaffected, it is unlikely that the removal of the negative charge leads to electrostatic imbalance within the protein, causing the observed phenotype. In contrast to the clear phenotype in ATOM complex biogenesis, the observed effects of the Salt E→A variant on kDNA segregation are minor, and can be explained by secondary effects due the failed ATOM complex as discussed earlier (5).

Collectively, our findings demonstrate that the structural integrity of the predicted TMH4-TMH5 GxxxG scaffold and its associated electrostatic network are indispensable for the membrane protein biogenesis function of pATOM36. To what extent the convergently evolved GxxxG motifs in OMM protein insertases in humans, such as MTCH1 and MTCH2, play a similar role remains an interesting question for future studies.

In this study, AlphaFold-based models were used to guide hypothesis generation and interpretation of experimental data. Structural features should therefore be considered approximate and not as experimentally validated atomic detail. The qualitative features described here were consistently observed across AlphaFold2 and AlphaFold3 models, but remain predictions.

## Experimental proedures

### Generation and characterization of structural protein models

The structural models of the proteins were predicted using the AlphaFold three server (23), computationally oriented into the planar OMM lipid bilayer using the PPM 3.0 web server tool available through the OPM database (24) and subsequently visualized with UCSF ChimeraX (version 1.10.1 ChimeraX) (50). The input sequences used for structural modeling included pATOM36 (Tb427.7.5700, residues 1–320), Mim1 (Q08176, residues 1–113), Mim2 (Q3E798, residues 1–87), MTCH1 (Q9NZJ7, residues 1–389), and MTCH2 (Q9Y6C9, residues 1–303).

Default parameters were applied for all methods unless stated otherwise. Salt bridge networks within the protein models were identified and analyzed using the ProteinTools web server hosted by the University of Bayreuth (51). Schematic figures were generated using BioRender (https://www.biorender.com).

### Transgenic cell lines

Transgenic procyclic cell lines, derived from *T. brucei* strain 427 or 29-13^5,53^, were cultured at 27 °C in SDM-79 medium supplemented with 10% (v/v) fetal calf serum (FCS). The pATOM36 (Tb427.7.5700) 3′UTR RNAi cell line was generated by Käser *et al.* (2016) and subsequently adopted for this study (5). Briefly, RNAi was performed using pLew100-derived stem loop vectors containing a blasticidin resistance gene, designed to target nucleotides 88 to 371 downstream of the pATOM36 stop codon. Tetracycline-inducible expression of the pATOM36 variants was achieved by cloning the corresponding full-length ORF, carrying the respective mutations, into a modified pLew100 expression vector containing a puromycin resistance gene and a C-terminal triple HA tag (52, 53).

### Digitonin extraction

Digitonin extraction was performed to obtain crude mitochondria-enriched fractions for assessing the mitochondrial localization of the protein of interest (38, 53). Procyclic cells were disrupted by resuspending 1 x 10^8^ cells in SoTe buffer (20 mM Tris-HCl, pH 7.5, 0.6 M sorbitol, 2 mM EDTA) containing 0.015% (w/v) digitonin. Following centrifugation (6800*g*, 4 °C), the mitochondria-enriched pellet was separated from the supernatant, and equal cell equivalents of each fraction were analyzed by SDS-PAGE and immunoblotting. In some cases, the mitochondria-enriched pellets were further subjected to alkaline carbonate extraction or detergent-based extraction.

### Alkaline carbonate extraction

Mitochondria-enriched fractions were resuspended in 100 mM Na_2_CO_3_ (pH 11.5). Half of each sample was retained as the total fraction. The remaining material was incubated on ice for 10 min and subjected to ultracentrifugation at 100,000*g* for 10 min at 4 °C. The resulting supernatant, containing soluble and peripheral membrane proteins, was collected, while the pellet, representing integral membrane proteins, was resuspended in 100 mM Na_2_CO_3_ (38). All fractions were subsequently analyzed by SDS-PAGE and immunoblotting. Blot imaging was performed using an Odyssey imaging system (LI-COR) employing near-infrared fluorescence detection in the preset 700 and 800 channels.

### BN-PAGE immunoblotting and quantification

Mitochondria-enriched fractions were solubilized on ice for 30 min in 20 mM Tris-HCl (pH 7.4), 50 mM NaCl, 10% glycerol, 0.1 mM EDTA, and 1 mM PMSF supplemented with 1% (w/v) digitonin. Following centrifugation at 16′000 x g for 15 min at 4 °C, the supernatants were resolved on 4 to 15% Mini-PROTEAN TGX Precast Protein Gels (Bio-Rad). Gels were subsequently incubated in SDS-PAGE running buffer (25 mM Tris, 1 mM EDTA, 190 mM glycine, 0.05% (w/v) SDS) to facilitate protein transfer onto the PVDF membrane (Thermo Scientific). Detection was performed using a HRP-conjugated secondary antibody. Signal development was achieved with the SuperSignal West Pico PLUS Chemiluminescent Substrate (Thermo Scientific). Chemiluminescent signals were subsequently visualized using a G-Box imaging system (Syngene). Complex band intensities and plot line profiles were analyzed using Fiji software (version 1.54p Windows) features (54).

### Immunofluorescence microscopy

Exponential cells (1 × 10^6^) were collected (2700*g*, 1 min, RT), washed with PBS, and resuspended in 50 μl PBS. Cells adhered to glass slides were permeabilized for 30 s in PBS with 0.2% Triton X-100, washed, and fixed in 4% paraformaldehyde for 10 min. After blocking in PBS with 2% BSA, cells were incubated sequentially with primary and secondary antibodies in PBS/2% BSA. Slides were washed, air-dried, and mounted with Vectashield containing DAPI (Vectorlabs). Imaging was performed on a Nikon Eclipse Ti2 Super-Resolution Microscope.

### kDNA area evaluation

kDNA area measurements were conducted on cells after 3 days of tetracycline induction. Procyclic cells were collected by low-speed centrifugation, washed with PBS, and allowed to adhere to glass slides. Adherent cells were fixed with 4% paraformaldehyde, and samples were mounted with Vectashield containing 4′,6-diamidino-2-phenylindole (DAPI) to visualize nuclear and kDNA (14). Z-stack images of fixed cells were acquired using a Nikon Eclipse Ti2 Super-Resolution Microscope with the preset 432 optical fluorescence channel. Quantification of kDNA areas was performed using Fiji software (version 1.54p windows) with a custom semi-automated macro. Briefly, Z-stacks near the focal plane were converted into maximum-intensity projections, followed by background subtraction to enhance signal-to-noise ratio. The images were then thresholded using a consistent intensity cutoff to generate binary masks, and kDNA areas were quantified using the “Analyze Particles” function. The kDNA loss was visually assessed, including only fully visible, intact cells.

### Antibodies

The following non-commercial antibodies were employed, with antibody dilutions specified for immunoblots (IB) and immunofluorescence (IF). Polyclonal rabbit pATOM36 (IB, 1:50), ATOM19 (IB, 1:50), ATOM46 (IB, 1:50), ATOM40 (IB, 1:10′000, IF, 1:1′000), and cytochrome *c* (IB, 1:1′000) have previously been described (5, 16, 20). The commercially available monoclonal antibodies used in this study were mouse anti-HA (Enzo Life Sciences AG, cat. no. CO-MMS-101 R-1000; IB, 1:5′000, IF, 1:1′000) and mouse anti-EF1α (Merck Millipore, cat. no. 05–235; IB, 1:10′000). Secondary antibodies for IB evaluation were IRDye 680LT goat anti-mouse, IRDye 800CW goat anti-rabbit (both LI-COR Biosciences; IB, 1:10′000), and HRP-coupled goat anti-rabbit (Sigma-Aldrich; IB, 1:4′000). Secondary antibody for IF was goat anti-rabbit Alexa Fluor 488 (ThermoFisher Scientific; IF 1:1′000) and goat anti-mouse Alexa Fluor Plus 594 (ThermoFisher Scientific; IF 1:1′000).

## Data availability

All data supporting the findings of this study are included in the article and its Supporting Information. Additional raw data and biological materials generated in this study are available from the corresponding author upon reasonable request.

## Supporting information

This article contains supporting information.

## Conflict of interest

The authors declare that there are no conflicts of interest with the contents of this article.

## References

[1] Archibald J.M. (2015). Endosymbiosis and eukaryotic cell evolution. Curr. Biol..

[2] Harsman A., Schneider A. (2017). Mitochondrial protein import in trypanosomes: expect the unexpected. Traffic.

[3] Lithgow T., Schneider A. (2010). Evolution of macromolecular import pathways in mitochondria, hydrogenosomes and mitosomes. Philos. Trans. R. Soc. B Biol. Sci..

[4] Lionaki E., Gkikas I., Tavernarakis N. (2016). Differential protein distribution between the nucleus and Mitochondria: implications in aging. Front. Genet..

[5] Käser S., Oeljeklaus S., Týč J., Vaughan S., Warscheid B., Schneider A. (2016). Outer membrane protein functions as integrator of protein import and DNA inheritance in mitochondria. Proc. Natl. Acad. Sci. U. S. A..

[6] Lionaki E., Gkikas I., Tavernarakis N. (2023). Mitochondrial protein import machinery conveys stress signals to the cytosol and beyond. BioEssays.

[7] Doan K.N., Grevel A., Mårtensson C.U., Ellenrieder L., Thornton N., Wenz L.S. (2020). The mitochondrial import complex MIM functions as main translocase for α-Helical outer membrane proteins. Cell Rep..

[8] Guna A., Stevens T.A., Inglis A.J., Replogle J.M., Esantsi T.K., Muthukumar G. (2022). MTCH2 is a mitochondrial outer membrane protein insertase. Science.

[9] Dimogkioka A.R., Elias A., Rapaport D. (2024). The mammalian protein MTCH1 can function as an insertase. bioRxiv.

[10] Schneider A. (2001). Unique aspects of mitochondrial biogenesis in trypanosomatids. Int. J. Parasitol..

[11] Cavalier-Smith T. (2010). Kingdoms Protozoa and Chromista and the eozoan root of the eukaryotic tree. Biol. Lett..

[12] Schneider A., Bursać D., Lithgow T. (2008). The direct route: a simplified pathway for protein import into the mitochondrion of trypanosomes. Trends Cell Biol..

[13] Schneider A., Ochsenreiter T. (2018). Failure is not an option – mitochondrial genome segregation in trypanosomes. J. Cell Sci..

[14] Stettler P., Schimanski B., Aeschlimann S., Schneider A. (2024). Molecular characterization of the permanent outer-inner membrane contact site of the mitochondrial genome segregation complex in trypanosomes. PLOS Pathog..

[15] Aeschlimann S., Kalichava A., Schimanski B., Berger B.M., Jetishi C., Stettler P. (2022). Single p197 molecules of the mitochondrial genome segregation system of *Trypanosoma brucei* determine the distance between basal body and outer membrane. Proc. Natl. Acad. Sci. U. S. A..

[16] Mani J., Desy S., Niemann M., Chanfon A., Oeljeklaus S., Pusnik M. (2015). Mitochondrial protein import receptors in Kinetoplastids reveal convergent evolution over large phylogenetic distances. Nat. Commun..

[17] Schneider A. (2018). Mitochondrial protein import in trypanosomatids: variations on a theme or fundamentally different?. PLOS Pathog..

[18] Desy S., Mani J., Harsman A., Käser S., Schneider A. (2016). TbLOK1/ATOM19 is a novel subunit of the noncanonical mitochondrial outer membrane protein translocase of *Trypanosoma brucei*. Mol. Microbiol..

[19] Pusnik M., Mani J., Schmidt O., Niemann M., Oeljeklaus S., Schnarwiler F. (2012). An essential novel component of the noncanonical mitochondrial outer membrane protein import system of trypanosomatids. Mol. Biol. Cell.

[20] Bruggisser J., Käser S., Mani J., Schneider A. (2017). Biogenesis of a mitochondrial outer membrane protein in trypanosoma brucei: targeting signal and dependence on a unique biogenesis factor. J. Biol. Chem..

[21] Vitali D.G., Käser S., Kolb A., Dimmer K.S., Schneider A., Rapaport D. (2018). Independent evolution of functionally exchangeable mitochondrial outer membrane import complexes. eLife.

[22] Popov-Čeleketić J., Waizenegger T., Rapaport D. (2008). Mim1 functions in an oligomeric form to facilitate the integration of Tom20 into the mitochondrial outer membrane. J. Mol. Biol..

[23] Abramson J., Adler J., Dunger J., Evans R., Green T., Pritzel A. (2024). Accurate structure prediction of biomolecular interactions with AlphaFold 3. Nature.

[24] Lomize A.L., Todd S.C., Pogozheva I.D. (2022). Spatial arrangement of proteins in planar and curved membranes by PPM 3.0. Protein Sci..

[25] Kumazaki K., Chiba S., Takemoto M., Furukawa A., Nishiyama K.I., Sugano Y. (2014). Structural basis of Sec-independent membrane protein insertion by YidC. Nature.

[26] Falk S., Ravaud S., Koch J., Sinning I. (2010). The C terminus of the Alb3 membrane insertase recruits cpSRP43 to the thylakoid membrane. J. Biol. Chem..

[27] Eaglesfield R., Tokatlidis K. (2021). Targeting and insertion of membrane proteins in Mitochondria. Front. Cell Dev. Biol..

[28] Hennon S.W., Soman R., Zhu L., Dalbey R.E. (2015). YidC/Alb3/Oxa1 family of insertases. J. Biol. Chem..

[29] Laskowski P.R., Pluhackova K., Haase M., Lang B.M., Nagler G., Kuhn A., Müller D.J. (2021). Monitoring the binding and insertion of a single transmembrane protein by an insertase. Nat. Commun..

[30] Nass K.J., Ilie I.M., Saller M.J., Driessen A.J.M., Caflisch A., Kammerer R.A., Li X. (2022). The role of the N-terminal amphipathic helix in bacterial YidC: insights from functional studies, the crystal structure and molecular dynamics simulations. Biochim. Biophys. Acta Biomembr..

[31] Senes A., Ubarretxena-Belandia I., Engelman D.M. (2001). The Cα—H⋅⋅⋅O hydrogen bond: a determinant of stability and specificity in transmembrane helix interactions. Proc. Natl. Acad. Sci. U. S. A..

[32] Waizenegger T., Schmitt S., Zivkovic J., Neupert W., Rapaport D. (2005). Mim1, a protein required for the assembly of the TOM complex of mitochondria. EMBO Rep..

[33] Dimmer K.S. (2012). A crucial role of Mim2 in the biogenesis of mitochondrial outer membrane proteins. J. Cell Sci..

[34] Fujiki Y., Hubbard A.L., Fowler S., Lazarow P.B. (1982). Isolation of intracellular membranes by means of sodium carbonate treatment: application to endoplasmic reticulum. J. Cell Biol..

[35] Aeschlimann S., Stettler P., Schneider A. (2023). DNA segregation in mitochondria and beyond: insights from the trypanosomal tripartite attachment complex. Trends Biochem. Sci..

[36] Stettler P., Aeschlimann S., Schimanski B., Schneider A. (2025). Assembly of the mitochondrial outer membrane module of the trypanosomal tripartite attachment complex. PLoS Pathog..

[37] Schnarwiler F., Niemann M., Doiron N., Harsman A., Käser S., Mani J. (2014). Trypanosomal TAC40 constitutes a novel subclass of mitochondrial β-barrel proteins specialized in mitochondrial genome inheritance. Proc. Natl. Acad. Sci. U. S. A..

[38] Käser S., Willemin M., Schnarwiler F., Schimanski B., Poveda-Huertes D., Oeljeklaus S. (2017). Biogenesis of the mitochondrial DNA inheritance machinery in the mitochondrial outer membrane of Trypanosoma brucei. PLoS Pathog..

[39] Hoffmann A., Käser S., Jakob M., Amodeo S., Peitsch C., Týč J. (2018). Molecular model of the mitochondrial genome segregation machinery in *Trypanosoma brucei*. Proc. Natl. Acad. Sci. U. S. A..

[40] Schimanski B., Aeschlimann S., Stettler P., Käser S., Gomez-Fabra Gala M., Bender J. (2022). p166 links membrane and intramitochondrial modules of the trypanosomal tripartite attachment complex. PLoS Pathog..

[41] Niemann M., Wiese S., Mani J., Chanfon A., Jackson C., Meisinger C. (2013). Mitochondrial outer membrane proteome of Trypanosoma brucei reveals novel factors required to maintain mitochondrial morphology. Mol. Cell Proteomics.

[42] Stojanovski D., Rissler M., Pfanner N., Meisinger C. (2006). Mitochondrial morphology and protein import—A tight connection?. Biochim. Biophys. Acta BBA - Mol. Cell Res..

[43] Povelones M.L., Tiengwe C., Gluenz E., Gull K., Englund P.T., Jensen R.E. (2013). Mitochondrial shape and function in trypanosomes requires the outer membrane protein, TbLOK1. Mol. Microbiol..

[44] Gerber M., Suppanz I., Oeljeklaus S., Niemann M., Käser S., Warscheid B. (2023). A Msp1-containing complex removes orphaned proteins in the mitochondrial outer membrane of *T brucei*. Life Sci. Alliance.

[45] Teese M.G., Langosch D. (2015). Role of GxxxG motifs in transmembrane domain interactions. Biochemistry.

[46] Barlow D.J., Thornton J.M. (1983). Ion-pairs in proteins. J. Mol. Biol..

[47] Kumar S., Nussinov R. (2002). Close-range electrostatic interactions in proteins. ChemBioChem.

[48] Russ W.P., Engelman D.M. (2000). The GxxxG motif: a framework for transmembrane helix-helix association1. J. Mol. Biol..

[49] Anderson S.M., Mueller B.K., Lange E.J., Senes A. (2017). Combination of Cα–H Hydrogen bonds and van der waals packing modulates the stability of GxxxG-mediated dimers in membranes. J. Am. Chem. Soc..

[50] Meng E.C., Goddard T.D., Pettersen E.F., Couch G.S., Pearson Z.J., Morris J.H., Ferrin T.E. (2023). UCSF ChimeraX : tools for structure building and analysis. Protein Sci..

[51] Ferruz N., Schmidt S., Höcker B. (2021). ProteinTools: a toolkit to analyze protein structures. Nucleic Acids Res..

[52] Wirtz E., Leal S., Ochatt C., Cross G.A. (1999). A tightly regulated inducible expression system for conditional gene knock-outs and dominant-negative genetics in Trypanosoma brucei. Mol. Biochem. Parasitol..

[53] Bochud-Allemann N., Schneider A. (2002). Mitochondrial substrate level phosphorylation is essential for growth of procyclic Trypanosoma brucei. J. Biol. Chem..

[54] Dewar C.E., Oeljeklaus S., Wenger C., Warscheid B., Schneider A. (2022). Characterization of a highly diverged mitochondrial ATP synthase Fo subunit in Trypanosoma brucei. J. Biol. Chem..

