# Supplementary material for: Disruption of GxxxG motifs in pATOM36 impairs biogenesis of the mitochondrial protein translocase of the outer membrane in Trypanosoma brucei
Source: J Biol Chem. 2026 May 6;302(6):113126. doi: 10.1016/j.jbc.2026.113126 (PMC13254594; doi:10.1016/j.jbc.2026.113126)
Supplement: Supplementary Figures [file mmc1.pdf]

Supplementary Information

Disruption of GxxxG motifs in pATOM36 impairs biogenesis of the mitochondrial protein translocase of the outer membrane in *Trypanosoma brucei*

Stephan Berger[1,2], Siri Speck[1], André Schneider*[1], and Christoph von Ballmoos*[1]

[1]Department of Chemistry, Biochemistry and Pharmaceutical Sciences, University of Bern, Bern, Switzerland.
[2]Graduate School for Cellular and Biomedical Sciences, University of Bern, Bern, Switzerland.

*Correspondence to: andre.schneider@unibe.ch, Christoph.vonballmoos@unibe.ch

Supplementary Figure 1:

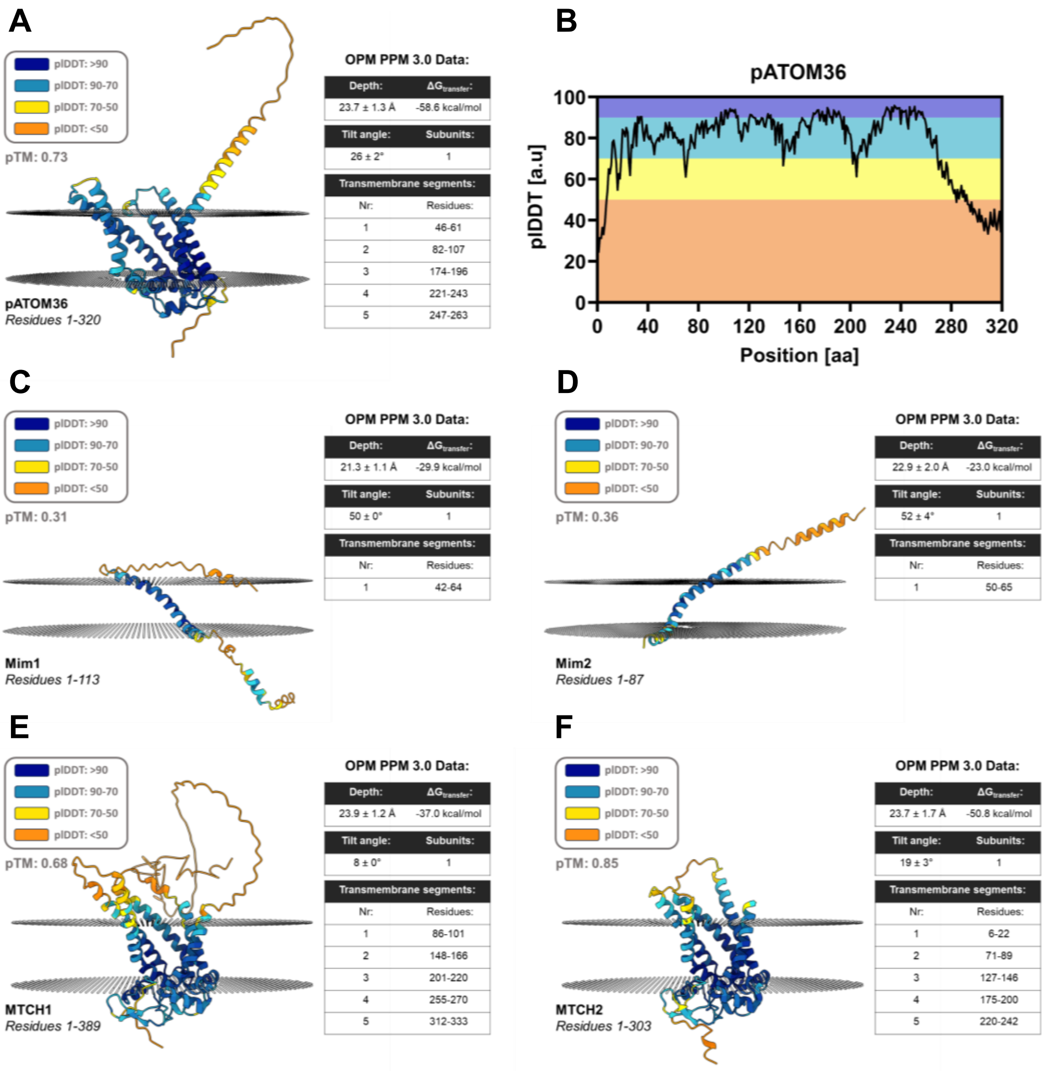

Supplementary Figure 1: AlphaFold3 models of outer mitochondrial membrane protein biogenesis enzymes with confidence scores and positioning. The structural models generated are shown with coloring corresponding to AlphaFold3 confidence scores, illustrating regions of high and low structural reliability. Residues with plDDT >90 are shown in dark blue, between 90 and 70 in light blue, between 70 and 50 in yellow, and residues with plDDT <50 in orange. The pTM values are indicated. The orientation and insertion of each model within the OMM were analyzed using the OPM PPM 3.0 server, providing parameters that describe membrane positioning, including depth/hydrophobic thickness, tilt, and the free energy of transfer ($\Delta G_{transfer}$). **A)** Model of wild-type pATOM36 (Tb427.7.5700). **B)** Graph depicting the per-residue plDDT values for the pATOM36 wild-type model **C)** Model of *S. cerevisiae* Mim1 (Q08176). **D)** Model of *S. cerevisiae* Mim2 (Q3E798). **E)** Model of human MTCH1 (Q9NZJ7). **F)** Model of human MTCH2 (Q9Y6C9).

Supplementary Figure 2:

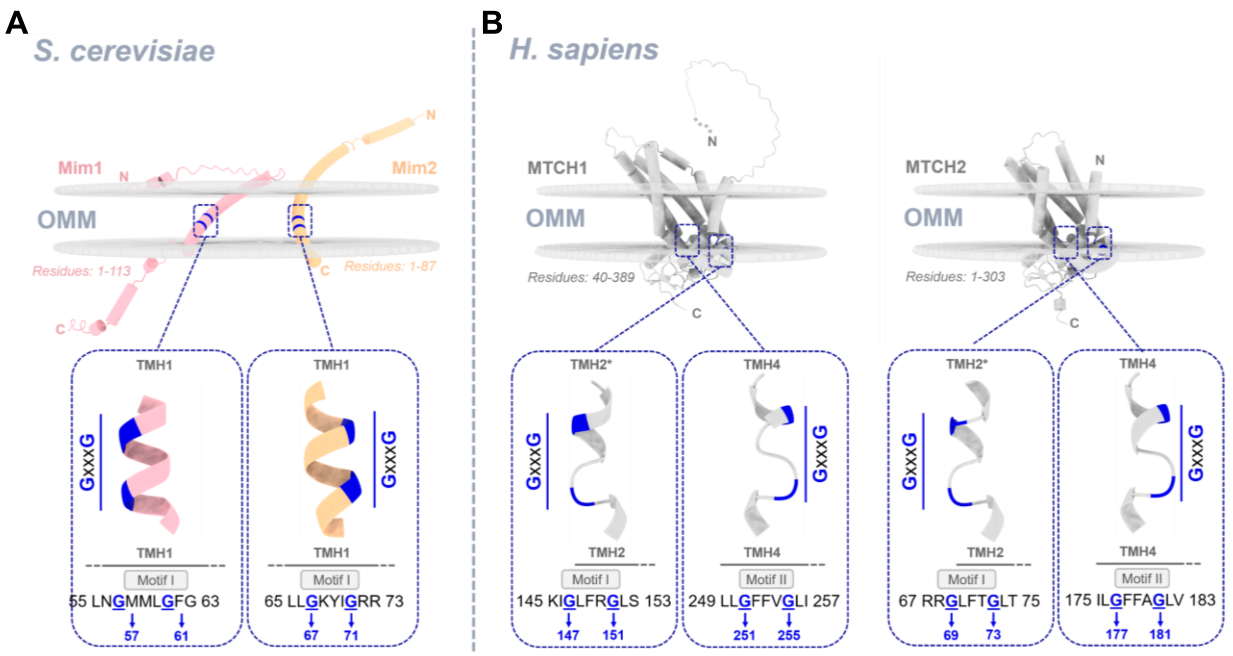

Supplementary Figure 2: Localization of transmembrane GxxxG helix-helix interaction motifs within the predicted models of yeast and human membrane protein biogenesis enzymes. Structural models and membrane positioning were calculated using AlphaFold3 and the PPM 3.0 algorithm, respectively. **A)** *S. cerevisiae* Mim1 in pink (Residues: 1-113) and Mim2 in orange (Residues: 1-87) embedded in the OMM (grey) with the transmembrane GxxxG motif (dark blue) **B)** Human MTCH1 (Residues: 74-389) and MTCH2 (Residues 1-303) in grey with transmembrane GxxxG motifs (dark blue).

Supplementary Figure 3:

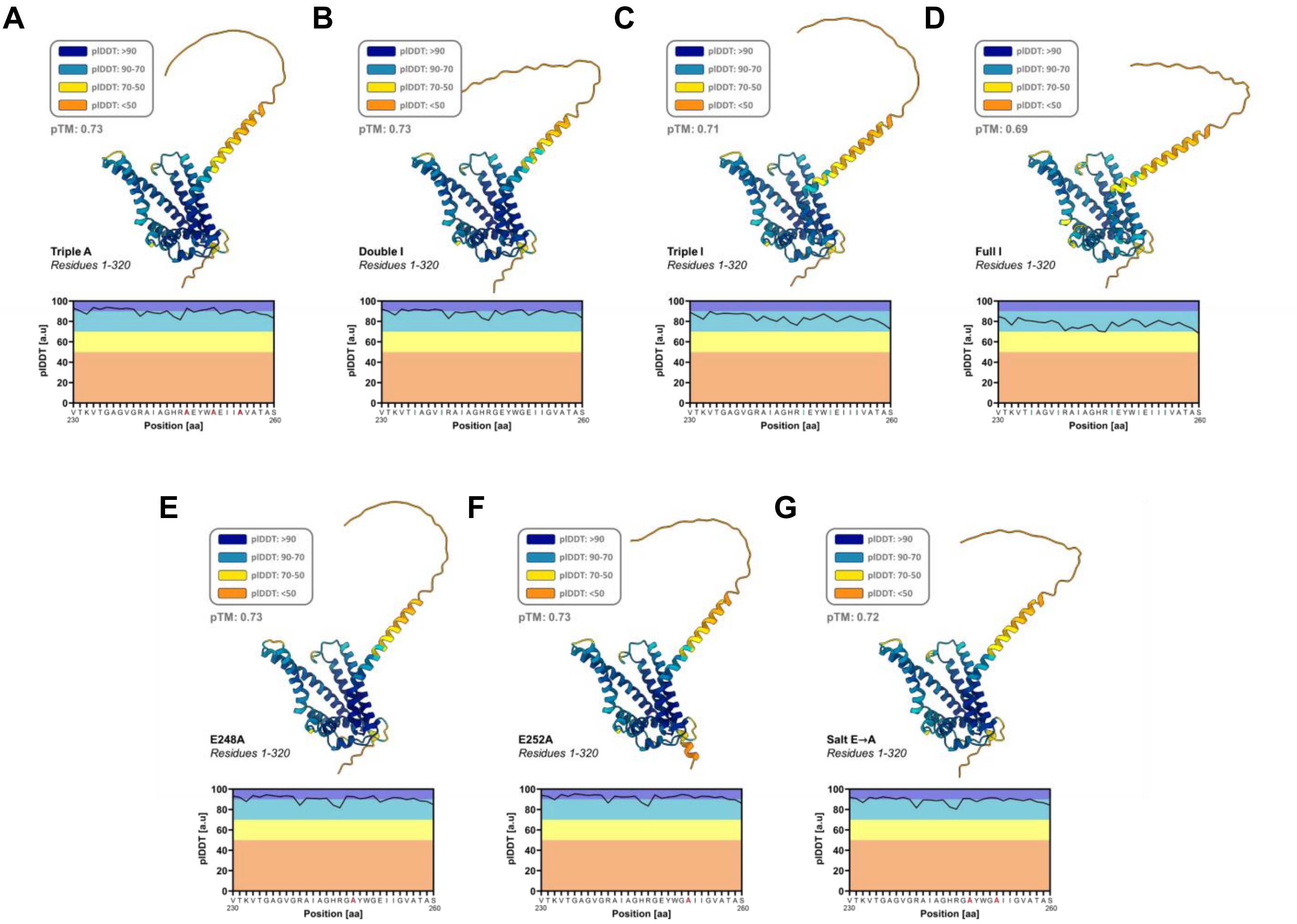

Supplementary Figure 3: AlphaFold3 models of the pATOM36 variants with prediction confidence values. AlphaFold3 structural models of pATOM36 variants generated in this work, colored according to the AlphaFold confidence metric. All mutants were derived from the pATOM36 Wild-Type sequence (Tb427.7.5700). The pTM values are indicated for each model. Each structure is accompanied by a graph showing the per-residue plDDT values for the mutated region from residues 230 to 260. Residues with plDDT >90 are shown in dark blue, values between 90 and 70 in light blue, between 70 and 50 in yellow, and residues with plDDT <50 in orange. **A)** Model of the Triple A variant. **B)** Model of the Double I variant. **C)** Model of the Triple I variant. **D)** Model of the Full I variant. **E)** Model of the E248A variant. **F)** Model of the E252A variant. **G)** Model of the Salt E→A (E248A, E252A) variant.

Supplementary Figure 4:

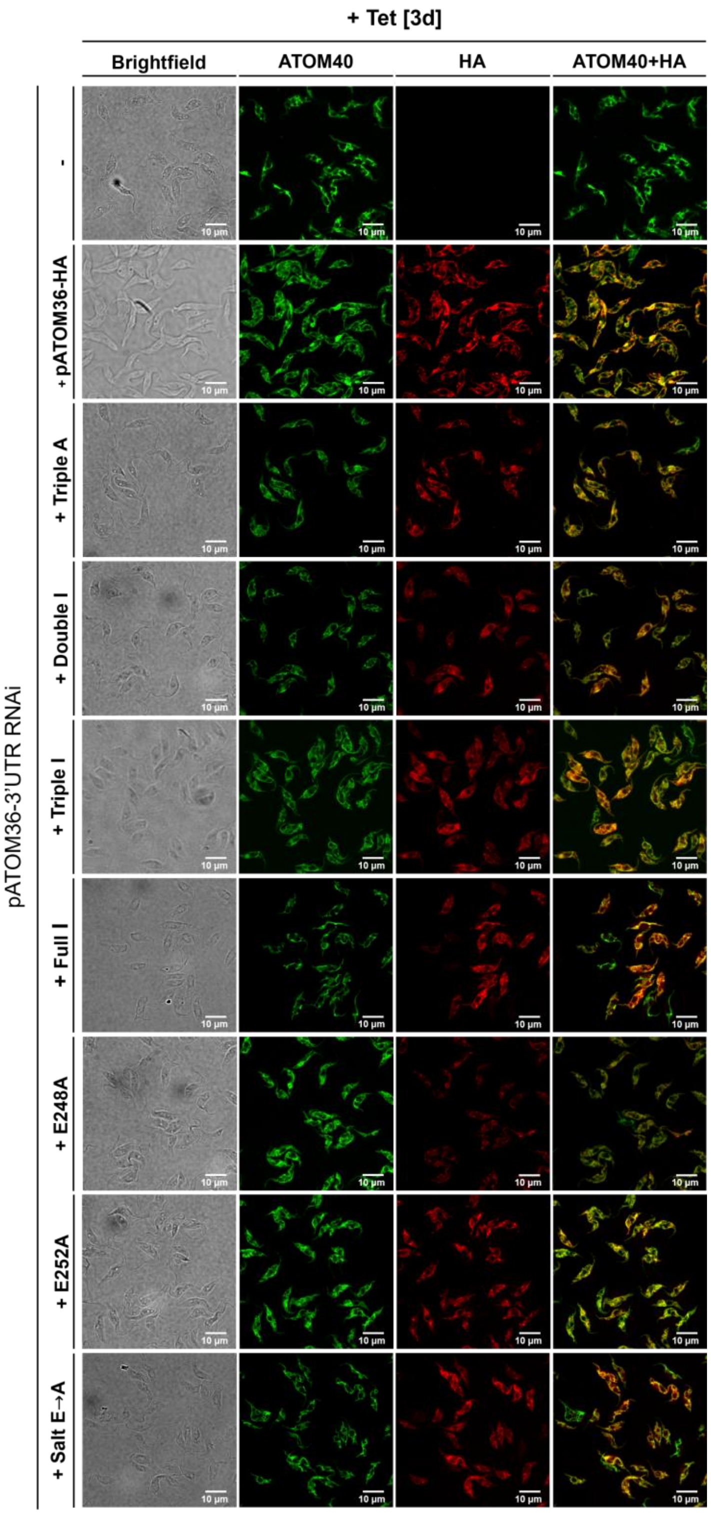

Supplementary Figure 4: Immunofluorescence analysis of established procyclic *T. brucei* cell lines. Immunofluorescence analysis of the indicated pATOM36 variant cell lines, including the pATOM36-3'UTR RNAi background cell line as a control, was performed 3 days after Tet induction. Widefield images were acquired in addition to confocal images. Confocal imaging was performed in the SD515 channel for ATOM40 (green) and in the SD641 channel for the HA epitope (red). Colocalization of ATOM40 and HA is illustrated in the merged image (yellow). The scale bar represents 10 µm.

Supplementary Figure 5:

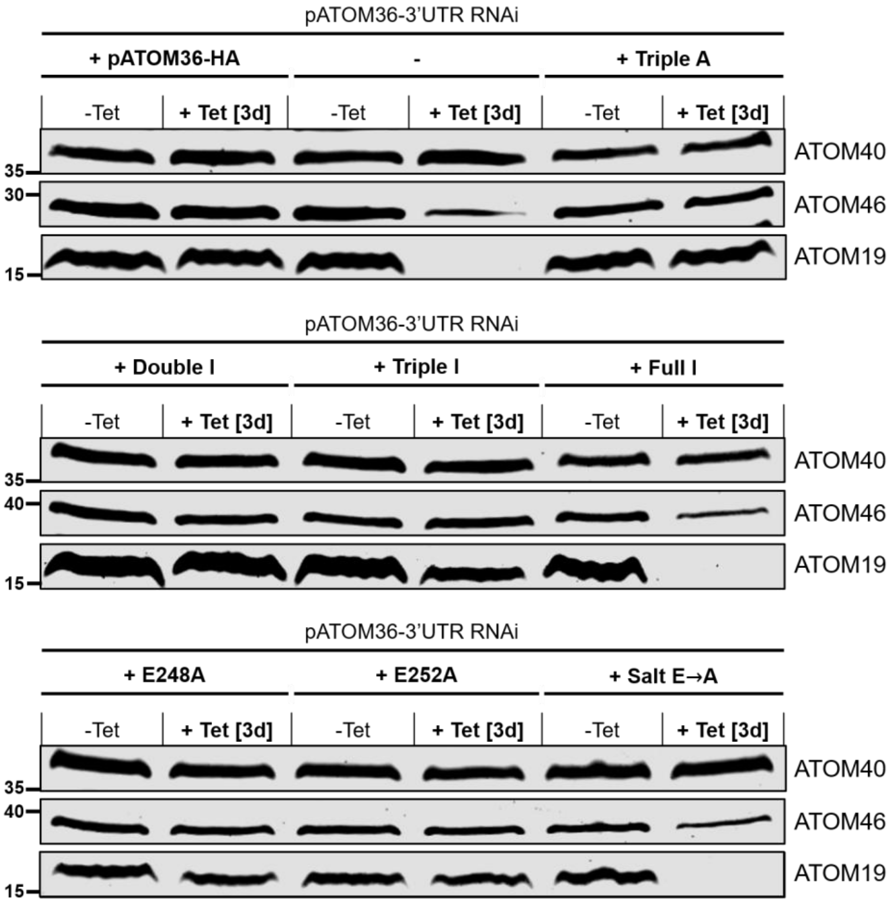

Supplementary Figure 5: Analysis of mitochondrial ATOM46 and ATOM19 levels in pATOM36 variant cell lines. Mitochondrial levels of ATOM46 and ATOM19 were analyzed in the indicated pATOM36 variant cell lines and, as a control, in the pATOM36-3'UTR cell line. Mitochondrial extracts from -Tet and +Tet (day 3) cells were examined. The β-barrel OMM protein ATOM40, which is not a substrate of pATOM36, served as a quantitative control. The α-helical OMM proteins ATOM46 and ATOM19 represent potential substrates of pATOM36.

Supplementary Figure 6:

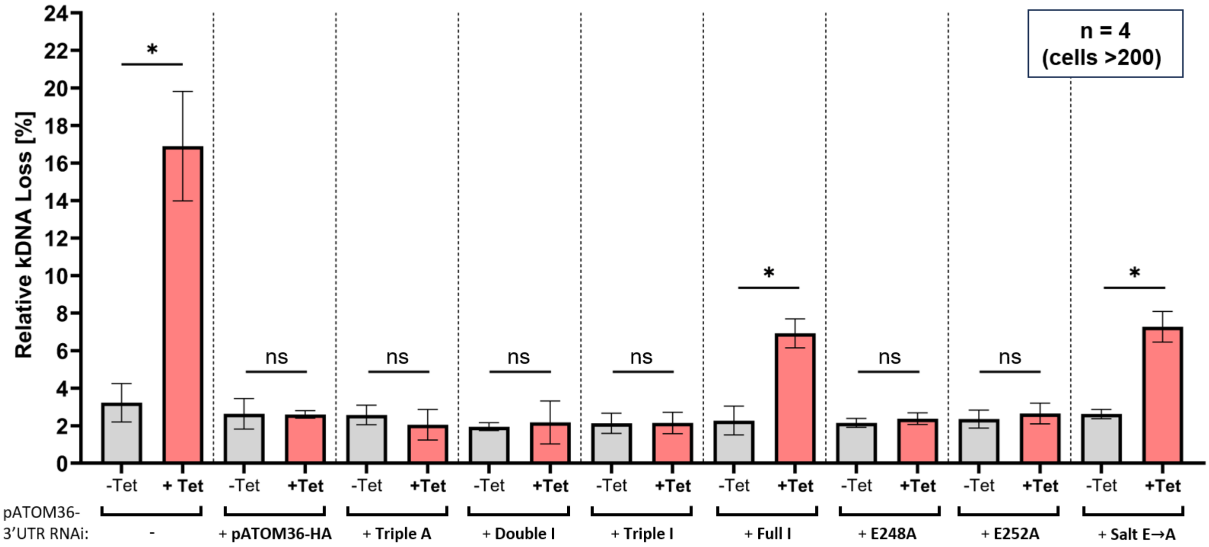

Supplementary Figure 6: Statistical evaluation of kDNA loss from microscopy images. Individual microscopy images (n = 4 per condition) were analysed independently. For each image, the percentage of cells lacking kDNA was quantified, with more than 200 cells evaluated per condition. Statistical comparisons between non-induced (-Tet) and day three post tetracycline induction (+Tet) groups were performed using a two-tailed Mann–Whitney test (95% confidence level). P values were evaluated in GraphPad Prism, with significance defined as ns ($p \geq 0.05$), * ($p < 0.05$), ** ($p < 0.01$), *** ($p < 0.001$), and **** ($p < 0.0001$). -Tet (grey) and +Tet (red) conditions are depicted, with the mean shown and error bars representing the standard deviation (SD) for each condition.

Supplementary Figure 7:

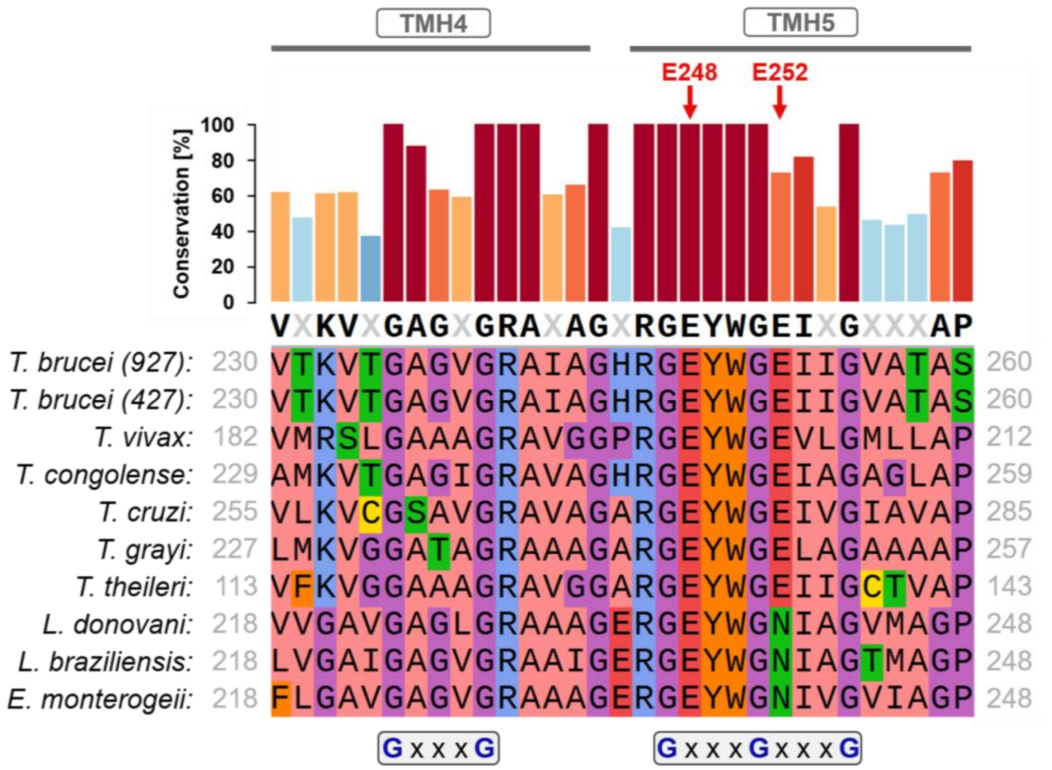

Supplementary Figure 7: Sequence alignment of GxxxG motifs containing region in pATOM36 orthologues
Shown is the MUSCLE amino acid sequence alignment of pATOM36 orthologs across various kinetoplastids. The individual amino acids within the alignment are colored according to their physicochemical properties (Zappo). The position of the structurally predicted TMHs 4 and 5 are highlighted in gray. E248 on TMH4 is fully conserved, while E252 shows partial conservation, limited to trypanosomes (marked in red). The GxxxG scaffold is fully conserved within pATOM36 orthologs.
